# Using Deep Learning Models to Predict Prosthetic Ankle Torque

**DOI:** 10.3390/s23187712

**Published:** 2023-09-06

**Authors:** Christopher Prasanna, Jonathan Realmuto, Anthony Anderson, Eric Rombokas, Glenn Klute

**Affiliations:** 1 Center for Limb Loss and Mobility, Seattle, WA 98108, USA; prasac@uw.edu (C.P.); ajanders@uw.edu (A.A.); 2 Department of Mechanical Engineering, University of Washington, Seattle, WA 98195, USA; rombokas@uw.edu; 3 Bionic Systems Lab, University of California, Riverside, CA 92521, USA; jrealmut@ucr.edu; 4 Department of Electrical and Computer Engineering, University of Washington, Seattle, WA 98195, USA

**Keywords:** biomechanics, machine learning, deep neural networks, robotic ankle prosthesis

## Abstract

Inverse dynamics from motion capture is the most common technique for acquiring biomechanical kinetic data. However, this method is time-intensive, limited to a gait laboratory setting, and requires a large array of reflective markers to be attached to the body. A practical alternative must be developed to provide biomechanical information to high-bandwidth prosthesis control systems to enable predictive controllers. In this study, we applied deep learning to build dynamical system models capable of accurately estimating and predicting prosthetic ankle torque from inverse dynamics using only six input signals. We performed a hyperparameter optimization protocol that automatically selected the model architectures and learning parameters that resulted in the most accurate predictions. We show that the trained deep neural networks predict ankle torques one sample into the future with an average RMSE of 0.04 ± 0.02 Nm/kg, corresponding to 2.9 ± 1.6% of the ankle torque’s dynamic range. Comparatively, a manually derived analytical regression model predicted ankle torques with a RMSE of 0.35 ± 0.53 Nm/kg, corresponding to 26.6 ± 40.9% of the ankle torque’s dynamic range. In addition, the deep neural networks predicted ankle torque values half a gait cycle into the future with an average decrease in performance of 1.7% of the ankle torque’s dynamic range when compared to the one-sample-ahead prediction. This application of deep learning provides an avenue towards the development of predictive control systems for powered limbs aimed at optimizing prosthetic ankle torque.

## 1. Introduction

Use of a practical model that maps from control commands and easily observed states to a controllable future state of a system, such as the torque about a powered ankle–foot prosthesis (PAFP), has the potential to improve the mobility of individuals with lower limb loss [1]. By simulating PAFP dynamics over a period of time for different control commands, a model can provide information to search for a sequence of control commands that achieve a desired prosthesis behavior, such as time-varying impedance parameters or terrain-specific angle-torque profiles. Real-time model predictive control (MPC) has been proposed to control assistive robotic devices during physical interaction with humans, allowing the robot to adapt its behavior [2,3]. Integrating MPC with real-time feedback from sensors into PAFP control schemes may enable the prosthesis to better adapt to the user’s movements, allowing for more natural and intuitive interactions between the user, device, and environment. A key benefit of this approach is the optimization of robot behavior over a finite time horizon by finding the optimal control inputs that minimize a cost function. This allows a controller to achieve a desired task, such as tracking a desired angle-torque profile while rejecting disturbances and minimizing energy consumption. Additionally, MPC can be designed to ensure the robot operates within the constraints of the system, such as joint limits and actuator saturation. For prosthetic applications, such constraints can be essential for safe and robust operation.

In terms of modeling PAFPs, control commands (e.g., motor current) can be mapped to motor torque for some devices including series elastic actuators. For parallel elastic actuators, the passive dynamics play a critical role in the total ankle torque (i.e., the total prosthetic ankle torque is the summation of the active and passive components). In addition to the passive elements of the prosthesis, there are other important factors, including variations in limb loading, shoe/keel mechanics, the dynamics of the other limbs and joints, and environmental conditions that can influence prosthetic ankle torque. Furthermore, even under steady-state walking conditions, stride-to-stride variations are inevitable, and it is important for prosthesis controllers to compensate for this variability [4].

Developing a method to predict prosthetic torques could better allow a PAFP controller (e.g., model predictive controller) to adapt to these factors that are unaccounted for in modern prosthetic control strategies [5]. This paper investigates different predictive model architectures with the goal of accurately predicting ankle torque over both short (one sample period or 33 ms) and long (twenty sample periods or approximately half a gait cycle) time periods. The results demonstrate the capability of high-fidelity predictive models and how they could be used in future prosthetic control systems.

### 1.1. The Role of System Models for Robotic Controllers and the Need for Better Modeling Approaches

**Figure 1:**
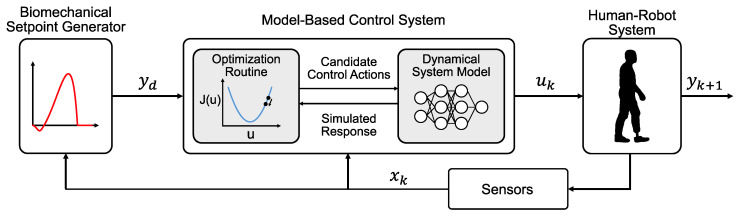
Block diagram of the architecture of a generic model-based prosthesis control system. A trajectory generator outputs a desired ankle torque $y_{d}$, which is then fed into an optimizer. The optimizer samples different possible control commands $u_{k}$ and conducts forward simulations based on system model predictions. The optimizer uses the results to determine which control command would achieve the closest one-sample ahead ankle torque response $y_{k+1}$ to the desired behavior. This control command is then sent to the human–robot system and the measurements of the system $x_{k}$ (e.g., loading and motion information from wearable sensors) are fed back to the optimizer for the next control sample.

A dynamical model of a robot prosthesis system would enable the use of model-based control methods, which use forward predictions to adjust control commands that drive the system toward a desired behavior (Figure 1). Model-based controllers that provide a distribution of future states (e.g., prosthetic ankle torque profiles) and their costs (e.g., the error between desired ankle torque profile and the predicted torque profile) based on candidate control commands are, when presented with limited environmental interactions, typically more sample-efficient and converge (i.e., minimize cost) more quickly than model-free techniques [6,7]. This is because a model-based controller is able to leverage the system model to focus the control command search space [8]. Model-free control strategies require more time to minimize cost (e.g., require more walking trials to train the controller and achieve a desired torque profile). In some cases, a model-based control law can be used as an initialization for a model-free learner (e.g., [9]). The model-free learner can then fine-tune the control law to overcome any model uncertainties. Despite their promise, model-based control systems have not been implemented in robotic prosthesis control because modeling human prosthesis dynamical behavior remains a challenge.

Deep neural networks (DNNs) provide a promising modeling strategy for model-based control of prosthetic ankles and are state of the art in other challenging learning tasks, such as natural language processing [10] and computer vision [11] that also involve high-dimensional nonlinear relationships. Once a DNN is sufficiently trained to model the human–robot system, it can be deployed to a real-time prosthesis control system and map system state measurements to an output prediction (e.g., ankle torque response), all without explicit knowledge of the system’s physics.

The capability of DNNs to output long-term predictions presents another key advantage in the context of prosthetic control. In certain cases, modeling approaches based on physics or first principles can provide insight toward system input–output relationships. However, it is often challenging and time-consuming to characterize system behaviors using first principles for systems with nonstationary dynamics that are highly nonlinear and vary over time (e.g., a human-prosthesis system). When mathematical process models are not known, DNN architectures have demonstrated an ability to accurately predict long-term behaviors including robotic trajectory [12] and human behavior prediction [13]. If high-fidelity DNN models that predict human-prosthetic dynamics can be developed, then it would be possible to proactively adjust prosthesis control actions to achieve a desired long-term trajectory or prepare for gait transitions.

### 1.2. Current Data-Driven Approaches in Prosthetic Control

Most data-driven biomechatronics research up to this point has been used to build models that relate environmental, system, and user data to intent recognition [14,15,16] or gait phase estimation [17] rather than to build predictive regression models of human–robot dynamical systems (e.g., estimating joint kinetics that cannot be observed instantaneously). A predictive model that outputs these dynamics directly can inform controllers and improve their performance (e.g., adapt the control inputs to achieve a desired ankle torque response). In addition, a model-based prosthesis controller can utilize joint dynamics information even outside the laboratory where motion capture (MoCap) is not available and inverse dynamics computations are more challenging.

Some early studies used neural networks and electromyography signal inputs to predict ankle dynamics; however, their predictions were noisy and less accurate when compared to a muscle model [18,19]. One explanation is that their shallow neural network architecture did not take the dynamic spatial–temporal relationships of human–robot systems into account. More recently, studies have successfully used more advanced neural network classes such as recurrent neural networks (RNNs) [20] and attention-based long short-term memory networks (LSTMs) [21,22] to generate reference trajectories for prosthesis controllers based on able-bodied data. Others have used wearable sensors and machine learning models to predict joint moments for exoskeleton control, also using able-bodied data [23]. However, deep learning models have not been used to predict prosthesis joint dynamics directly for individuals with transtibial amputation.

### 1.3. Deep Learning Modeling Approach

The field of wearable robotics is increasingly incorporating deep learning to improve control systems and user adaptability [24]. Recent studies have applied deep learning for intuitive control of powered knee–ankle prostheses [25], estimating joint moments and ground reaction forces in various walking conditions [26], and predicting joint moments in real time for exoskeleton controllers [27,28]. This signifies a growing trend towards more responsive and adaptive assistive technologies, yet gaps remain in understanding the potential and limitations of deep learning methods for PAFP control.

The development of models that can predict ankle mechanics is a key milestone in implementing a model-based control strategy for prosthetic devices, and this paper investigates different model architectures for predicting prosthetic ankle torques. The models are trained and evaluated based on how well they can predict prosthetic ankle torque values from previously collected data. These methods use only a small number of model input features and do not rely on a full-body MoCap suit or a large array of electromyography sensors. Experimental data in the form of total prosthetic ankle torques from optical MoCap (target outputs) and system state data (predictor inputs) from optical MoCap, force plate measurements, and on-board sensors are used to train the predictive models.

The output of the models was chosen to be the forward predictions of the prosthetic ankle torque. These output targets were computed using the full-body MoCap marker set (Vicon) with inverse dynamics principles to derive the total torque value at the ankle based on the movements of all anatomical structures that affect the joint. The reason for choosing the prosthetic ankle torque as the time series of interest is because many robotic prosthesis control methods aim to achieve a desired ankle torque or deploy a mathematical formula based on ankle impedance (i.e., prescribing an assistive ankle torque based on changes in ankle position).

Three DNN architectures were developed and trained: (i) a simple multilayer feedforward network (FFN), which passes information in one direction [28]; (ii) a gated recurrent unit network (GRU), which includes feedback connections and gates used to keep track of long-term dependencies in the input sequences [29]; and (iii) a dual-stage attention-based gated recurrent unit network (DA-GRU), which shares the capabilities of the GRU and adds attention mechanisms that assign weights to the different elements of the input sequence based on their relevance to learning the given task [30]. Each DNN was trained to predict ankle torques from inverse dynamics a short time period (one sample) into the future. The one-sample-ahead prediction performances of the DNNs and an analytical model of the PAFP system, which was derived based on first principles, are then directly compared to PAFP torque computed from MoCap and inverse dynamics. Additionally, we assess the long-term predictive capabilities of neural networks that could realistically be applied to prosthetic control. Each DNN was trained to predict twenty samples, approximately half a gait cycle, into the future to compare each DNN’s ability to predict prosthetic ankle torques. Half a gait cycle was chosen, as it could enable a prosthesis controller to proactively respond to stride-to-stride variations or gait transitions.

In summary, while most prosthetic applications have yet to incorporate predictive models, advanced feedback control systems stand to benefit significantly from them, especially for managing the complex human–robot interactions in prosthetic ankle–foot devices. One of the primary challenges in predicting prosthetic ankle torque stems from the fact that it is influenced by both the control action of the device and the variable kinematic pattern adopted by the user when using devices that incorporate parallel elastic elements. Traditional physics-based models struggle to account for these complexities, making them less effective for real-time control. The main contribution of this paper is the development and comparison of deep learning models that are specifically designed to predict this shared human–robot state, thus paving the way for more adaptive and intuitive prosthetic control systems in the future.

## 2. Materials and Methods

### 2.1. Prototype Ankle–Foot Prosthesis and Experimental Setup

**Figure 2:**
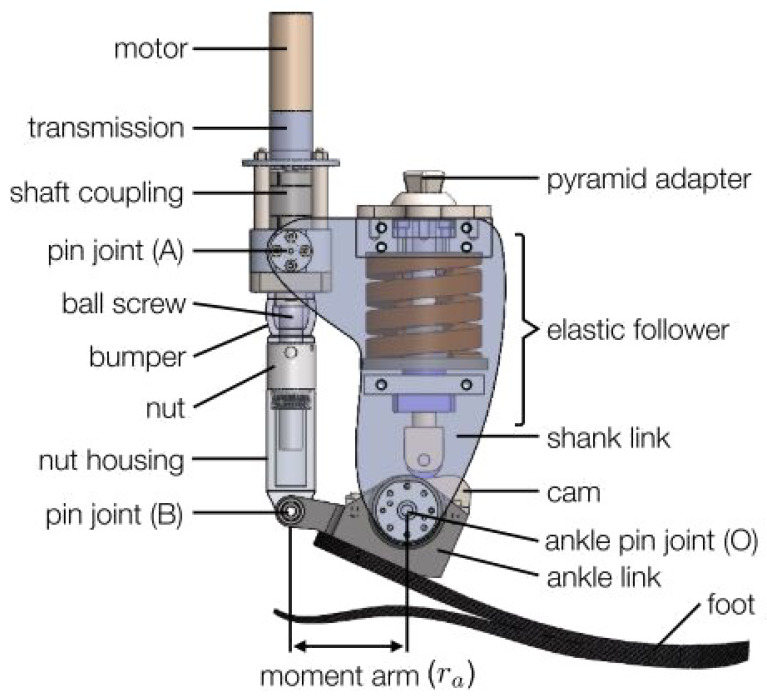
Illustrative rendering of the prototype PAFP with major components labeled (note that some components are transparent for ease in visualization).

A prototype PAFP was used in this research [31]. Figure 2 shows the prototype and all its subcomponents. A cam-based spring acts across the ankle joint and provides a nonlinear elastic response which mimics the elastic response of a biological ankle [31]. The spring acts in parallel to the powered drivetrain which provides active torque and consists of a motorized link acting across the shank and ankle links. A 12-bit capacitive encoder is attached to the prototype PAFP and is used to sense the angular position of the prosthetic ankle joint via a serial peripheral interface. Additionally, the motor current and velocity are transmitted by the motor drive to the custom embedded system’s analog-to-digital peripheral.

A pilot (N = 1) study was conducted where a subject performed steady-state walking at 1 m/s using the prototype PAFP. A safety harness was included in the experimental setup as any data collected when the subject touched the treadmill handrails were discarded. A novel human-in-the-loop symmetry learning strategy was used to control the PAFP, where an adaptive gain iterative learning control algorithm adjusts the PAFP’s torque after each walking trial to match the achieved intact ankle torque [32]. A total of 23 walking trials were conducted, with each trial having a unique control command trajectory and prosthetic ankle torque profile. The experimental setup consisted of a split-belt force-sensing treadmill (Bertec), a 12-camera MoCap system (Vicon), and a human subject donning the PAFP with custom embedded system and tethered power supply. Marker trajectories and ground reaction force (GRF) data were recorded at 120 Hz and 1200 Hz, respectively. The raw data were filtered using a digital, fourth-order, low-pass Butterworth filter with cutoff frequencies of 25, 6, 50, and 50 Hz for kinetics, kinematics, GRF, and embedded system signals (i.e., motor currents and velocities), respectively. A custom 15-segment whole body model was created in Visual 3D and the markers on the prosthetic limb mirrored the markers on the intact limb. For presentation purposes, GRF data were thresholded at 20 N for swing and stance segmentation. Joint angles were calculated using inverse kinematics and moments were calculated using standard inverse dynamics and normalized by the subject’s body mass. The prototype PAFP was fitted to the left leg of the subject, using their as-prescribed socket and suspension system, and aligned by a certified prosthetist. The subject provided written informed consent to participate in the experimental protocol, approved by the VA Institutional Review Board. The subject was a healthy, active, 85-kg, unilateral transtibial amputee. The data collected during this preliminary investigation were used to build the models in this study.

### 2.2. Baseline: Analytical Regression Model

An analytical least squares regression model was developed to provide a baseline comparison to the DNN models (see Appendix A). Similar to training a DNN, least squares regression attempts to fit a set of parameters to data. However, one advantage to least squares over deep learning is that there is always an analytical solution (i.e., a known set of optimal parameters).

The analytical regression model was derived based on the known physics of the prototype PAFP’s dynamics. For this system, the total prosthetic ankle torque $\tau_{p}$ is the sum of the passive torque from the parallel elastic element during loading $\tau_{l}$ and the active torque generated by the motor and ball screw $\tau_{a}$. The analytical regression problem is formulated as:

(1) $$\begin{array}{cc} \tau_{p}-\tau_{a} & =\tau_{l}\\ \tau_{p}-R_{\tau }\left(\theta \right)k_{\tau }i_{a} & =p_{3}\theta^{3}+p_{2}\theta^{2}+p_{1}\theta +p_{0} \end{array}$$

where $R_{\tau }\left(\theta \right)$ is the effective transmission and is a function of the prosthetic ankle angle θ. The rated motor torque constant provided by the motor manufacturer is denoted by $k_{\tau }$. The total prosthetic torque $\tau_{p}$ is computed using the MoCap system and full-body inverse dynamics. Additionally, the prosthetic ankle angle θ is the output of the PAFP-mounted encoder sensor. Similarly, the motor current command $i_{a}$ is sampled by the embedded system. The passive PAFP elements (e.g., cam device and spring) were designed and characterized in a previous study using a third-degree polynomial function whose parameters (i.e., $p_{0}$, $p_{1}$, $p_{2}$, and $p_{3}$ in Equation (1)) were fit via least squares from axial bench test data [31]. Preliminary work using these parameters resulted in high model prediction bias. Refitting these parameters using data from the human subject experiment sufficiently reduced this bias, presumably by capturing the as-worn conditions of the PAFP and the variability introduced by user behavior.

The prosthetic torque signal $\tau_{p}$ was time-shifted ahead by one sample relative to the prosthetic ankle angle θ and motor current command $i_{a}$ signals. All data were then divided using the train–test split method, where the last 15% of experimental walking trials were reserved to evaluate the analytical model. Note that the test dataset was equivalent to the dataset used to evaluate the DNN models discussed in Section 2.3. The remaining data were shuffled, and then used for model training and cross-validation. Five-fold cross-validation was implemented and the parameters of the best-performing model out of the five trained models were used for testing and evaluation.

### 2.3. Neural Network Architectures

While DNN training is typically more challenging when compared to least squares regression, the class of functions that can be learned effectively with a DNN is richer. Certain DNN architectures have mechanisms that assist in learning temporal dependencies within the data and others are capable of encoding the relative importance of each input features with regards to predicting the output variable.

**Figure 3:**
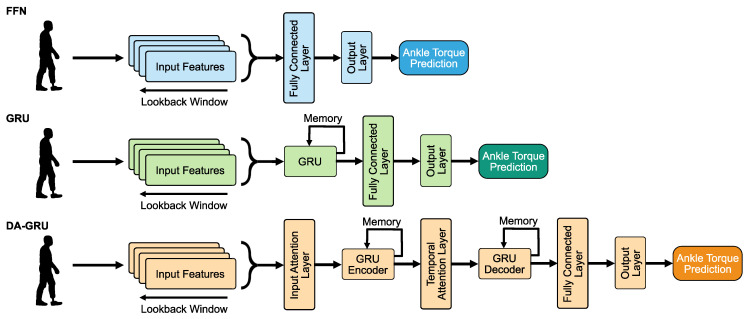
Visual illustration of the deep neural network architectures. The time history of input features are concatenated and fed into each network. Each DNN is trained to output a predicted PAFP ankle torque one timestep ahead or twenty timesteps ahead of the current timestep. Note that only one FFN and GRU are displayed in this diagram, but multiple layers were tested during hyperparameter optimization.

In this study, three DNN architectures were trained to predict prosthetic ankle torques from inverse dynamics (see Figure 3). Each DNN model was implemented within the PyTorch framework [33]. The following were used as the input features for the DNN models: left and right vertical GRF, prosthesis ankle encoder angles, prosthesis-side hip angles, motor current commands, and motor velocities.

#### 2.3.1. FFN

An FFN consists of a series of fully connected layers that connect every neuron in one layer to every neuron in the other layer [34]. This architecture was chosen because it is easy to implement and common across many applications. However, FFNs tend to not perform as well as more application-specific networks and cannot learn to modulate the feature inputs directly for better prediction results. Additionally, this type of network has no temporal memory since the layers connect unidirectionally (i.e., there are no cycles or feedback loops in the network).

#### 2.3.2. GRU

A multi-layered GRU network consists of recurrent layers and a hidden state at each timestep [29]. The GRU architecture was included in this study for its applications towards tasks related to learning complex spatial–temporal dependencies in time series data. At each timestep, the GRU layer adds information to or removes information from the hidden state, which makes it particularly effective for learning temporal dependencies in time series data. The GRU layer uses a reset gate which controls the level of state reset, update gate, which controls the level of state update, and candidate reset state, which controls the level of update added to hidden state. This network is similar to long short-term memory (LSTM) networks but has less parameters to train due to the lack of an output gate. However, the performance of GRUs have been shown to be similar to LSTMs for certain tasks [35] and can even outperform them for training on smaller and less frequent datasets [36].

#### 2.3.3. DA-GRU

A DA-GRU network, inspired by the DA-RNN [30], was developed for this study. This network uses a GRU architecture but also includes an attention mechanism that adaptively extracts the most relevant features at each timestep using an encoder-based hidden state [37]. An encoder is a type of network that maps input sequences into a representation where more relevant information is weighed more (i.e., it enhances and directs focus to the important parts of the input data). Similarly, a temporal attention mechanism is also used to decode the relevant encoder hidden states across timesteps. The DA-GRU was chosen because it not only has the ability to capture the long-term temporal dependencies of the dataset similar to the GRU, but can also adaptively select the most relevant input features across the dataset. In addition, the attention mechanisms help the optimizer escape from local minima to reach a better minimum loss and help avoid the vanishing gradient problem when dealing with sequential time series data.

### 2.4. Data Processing

Data sampled and logged from experimental trials were used to create the datasets for neural network training and evaluation. Once all data signals were filtered, the signals were then downsampled from 120 Hz to 30 Hz to reduce training time with minimal loss in performance since most human movement data information is contained within 15 Hz [38]. At this sampling rate and from the 23 30-s walking trials, a total of 20,700 data points per variable (i.e., input features and output target) were used for model training. For all models, the output target (i.e., ground truth) is the PAFP ankle torque computed from MoCap and inverse dynamics. The analytical regression model and the DNNs were trained to predict the PAFP ankle torque valued one sample (i.e., 33 ms) into the future. Additionally, DNN models were also trained to predict ankle torque values twenty samples ahead, which was approximately half a gait cycle into the future.

Each model input feature was scaled to the interval [0, 1] based on its maximum and minimum values within the training dataset. This procedure was conducted in order to transform each feature to a similar scale, which reduces the model’s sensitivity to magnitude shifts during learning and also prevents the features with large ranges in values from dominating predictions. A rolling lookback time window (i.e., sequence) was also implemented and defined how many feature values of previous timesteps were used for model predictions. The length of the lookback period was a tuneable hyperparameter and was adjusted to assist the DNNs in learning the time dependencies within the data. The data were then split into the three types of datasets commonly used in deep learning applications: training, validation, and testing. The first 70% of the data from each time series (i.e., walking trial) were used to create the training set, the following 15% were used to create the validation set, and the remaining 15% were used to create the test set. The training dataset was then split into batches and the batch size was a tuneable hyperparameter that was optimized.

### 2.5. Loss Function and Network Parameter Optimization

The loss function used during DNN training was chosen as the mean squared error (MSE) between the neural network output prediction and the measured target. The AdamW optimization algorithm [39], an expansion of the Adam optimization strategy with decoupled weight decay functionality, was used to update the network weights. A learning rate scheduler was also implemented, which allowed the dynamic learning rate to be reduced based on when validation predictions stopped improving [40]. The number of epochs with no improvement after which the learning rate reduced (i.e., patience) was set to 3. Thus, the optimizer would ignore the first two epochs with no improvement and only decreased the learning rate after the third epoch if the loss still had not improved. Furthermore, the number of epochs to wait before resuming normal operation after the learning rate has been reduced (i.e., cooldown) was set to 3. The factor by which the learning rate reduced γ was a tuneable hyperparameter. The minimum learning rate was set to 1 × 10${}^{-5}$.

### 2.6. Network Hyperparameter Optimization Procedure and Outcomes

The Optuna framework [41] was used to optimize the network training hyperparameters and a total of 500 combinations of hyperparameters were tested for each network (see Table 1). The hyperparameters included the sequence length, number of hidden layers, number of hidden units, dropout probability, batch size, initial learning rate, AdamW weight decay coefficient, and learning rate reduction factor. The DA-GRU hyperparameter optimization also included the number of hidden decoder units but excluded the dropout probability. Additionally, the number of hidden layers was fixed for DA-GRU hyperparameter optimization to match the dual-layer architecture of the DA-RNN network [30].

The performance of the model was evaluated on the validation set every epoch. To reduce the chance of overfitting, an early stopping protocol was used to take the validation loss and count the number of epochs since the loss improved. If the loss stopped decreasing for 10 epochs in a row, the training stopped and the best-performing model was saved. The maximum number of training epochs was set to 1000. Once all the hyperparameters were optimized for the three networks over the 500 Optuna trials, predictions were generated and evaluated on the test dataset.

### 2.7. Analysis

Root Mean Squared Error (RMSE) and the Pearson correlation coefficient (PCC) were used as measures to report the performance of the different models. RMSE is one of the most widely used measures for evaluating the quality of regression model predictions and it shows how far predictions fall from measured true values. Since the errors are squared before they are averaged, the RMSE gives a relatively high weight to large errors, which is useful in this application, since large prediction errors are undesirable. PCC is an analysis that measures the strength of association between two continuous signals and can indicate if there are any time shifts (i.e., lags) between them. This metric was chosen since actuation timing plays a key role in the context of PAFP controllers (e.g., personalization to each user’s unique biomechanics and changing prosthesis behaviors or impedance parameters at various phases of the gait cycle). In an absolute best-case scenario, the RMSE will be equal to 0 and PCC would be equal to 1. Additionally, percent RMSE values were used to compare the performance of the models. Percent RMSE was computed by dividing the RMSE value by the ankle torque’s range of values within the walking trial.

## 3. Results

**Figure 4:**
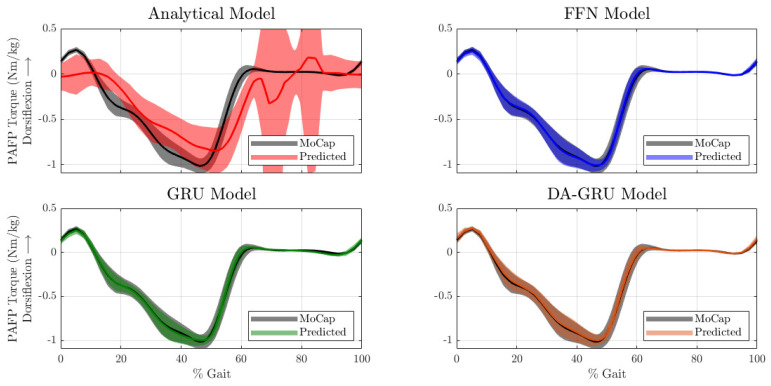
One-sample ahead model predictions of total PAFP torques across gait cycles. The periodic time series are time-normalized across the gait cycle for better visualization. The black time series data labeled as “MoCap” represents the PAFP ankle torque calculated using inverse dynamics. This data serve as the ground truth for model validation. The thin solid lines represent the mean and the corresponding shaded areas represent ±1 standard deviation.

**Figure 5:**
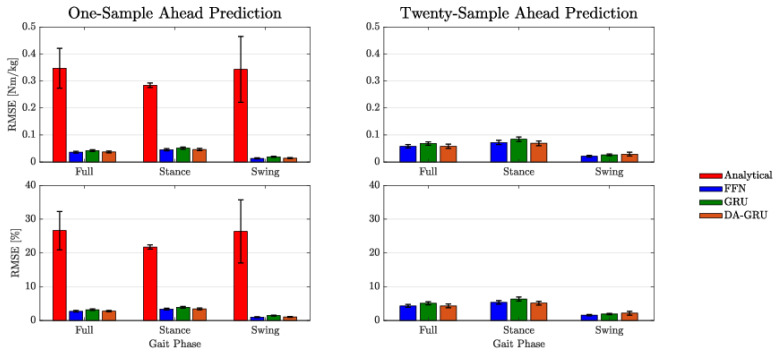
RMSE (**top** row) and RMSE percent error (**bottom** row) for each model class for both one-sample ahead (**left** column) and twenty-sample ahead (**right** column) predictions. RMSE percent error was calculated by dividing the RMSE value by the range of ankle torque values within the walking trial. Errors are shown for stance and swing phases individually as well as the full gait cycle. The error bars represent the standard error.

The prediction accuracy of the DNN models for the test dataset was much higher when compared to the analytical model (see Figure 4 and Figure 5). Note that the time series data shown in this section were used only for model evaluation and were not part of the training data. Furthermore, the desired torque setpoint was not included in Figure 4 because this signal only accounts for the active torque. The PAFP used in this experiment is a parallel elastic actuator, and therefore, the setpoint command only accounts for the drivetrain dynamics (i.e., active torque) but there are passive system components that also influence the total prosthetic ankle torque.

For both one-sample ahead and twenty-sample ahead prediction tasks, the mean RMSE of the results were generally within 5% of the total range of PAFP ankle torque values (see Table 2). When considering approximately two-thirds of the error data (i.e., mean ±1 standard deviation), DNN errors were only within 8% of the PAFP ankle torque’s dynamic range. The DNN errors were notably lower than the analytical model which had a mean RMSE of 26.6% of the PAFP ankle torque’s dynamic range. When including the ranges within one standard deviation of the mean, the error went up to 58% of the range of ankle torque values. In general, DNN prediction errors increased from one to twenty samples into the future; however, this increase was small and all errors were within the same order of magnitude. An analytical model was not derived for the twenty-sample ahead prediction problem, however, the twenty-sample ahead predictions of the DNN models outperformed the one-sample-ahead analytical model predictions by a substantial margin (see Figure 5 and Table 2).

DNN predictions showed a high PCC for both prediction tasks when compared to the analytical model (see Table 2). All PCC values for the DNNs were above 0.99 for the one-sample-ahead prediction task and only dropped by approximately 0.01 for the twenty-sample ahead prediction task. Compared to the analytical model, which only showed a mean PCC value of 0.82 with a standard deviation close to 25% of its mean, the DNN results demonstrate a much higher correlation to the desired target predictions. Similar to the RMSE results, the PCC of the twenty-sample ahead DNN predictions consistently outperformed the PCC of the one-sample-ahead analytical model predictions, which further demonstrates the long-term predictive capabilities of DNN models. Finally, the model training times were as follows: 0.05 s for the analytical regression model, 43 s for the FFN, 2 min 15 s for the GRU, and 13 min 18 s for the DA-GRU. Note that these training times are based on the DNNs with their optimized hyperparameters.

## 4. Discussion

This study developed predictive models to enable future predictive controller development for prosthetic devices, which could become feasible outside the laboratory and without relying on a full-body MoCap suit or a large array of wearable sensors. The prosthetic torque predictions can inform prosthesis controllers, help anticipate changes in dynamics, and proactively actuate the ankle to achieve a desired behavior. Across all the analyzed metrics, the FFN, GRU, and DA-GRU models (i.e., DNN models) outperformed a nominal analytical regression model. An interesting discovery was that all DNNs demonstrated approximately the same performance (<0.01 Nm/kg difference in RMSE, <0.75% difference in percent RMSE, and <0.01 difference in PCC). Additionally, the results show that all DNNs can be retrained to predict approximately half a gait cycle into the future without compromising overall performance. When compared to the one-sample-ahead predictions, the twenty-sample ahead prediction performance for all DNNs only decreased by an average of 0.023 Nm/kg and 1.7% of the PAFP ankle torque’s dynamic range. The decrease in the PCC value across all DNNs was also small at approximately 0.01. Figure 5 displays the approximately equivalent results for all the DNNs and how the predictions errors are within one standard deviation of each other. Due to the simpler architecture and faster training time, it may be more beneficial to use the FFN for deployment onto embedded systems.

Overall, the DNN model prediction results were more accurate than the nominal analytical regression model. Additionally, the DNNs were able to generalize across the whole gait cycle, while the nominal analytical model produced very poor results during the swing phase. This strategy, therefore, eliminates the need to design hybrid system models and finite state machines, thus reducing the amount of control parameters that need to be tuned by experts. Additionally, this method eliminates the time-intensive process of characterizing the mechanics of prototype PAFPs via analytical methods (e.g., deriving physics-based equations of motion, mechanical testing, mechanical rig construction, parameter fitting, etc.). Instead, PAFP researchers only need to collect preliminary walking data and use that data to train the DNNs. The DNNs also demonstrated the ability to predict future behavior with only a small loss in performance. This unique quality is not shared by analytical or physics-based models and could enable model predictive controllers for PAFPs.

Even though the analytical regression model was designed based on the most dominant and identifiable features of the PAFP system’s dynamics, its accuracy was limited. The low accuracy of the analytical regression model could be due to the many factors other than ankle kinematics and drivetrain dynamics that can influence prosthetic ankle torque (e.g., variations in limb loading and the dynamics of proximal joints). Even with a clear understanding of the prosthetic drivetrain, cam profile, and passive spring, the analytical regression model cannot account for unmeasured factors and thus, fails to properly characterize prosthetic ankle torque. On the other hand, the DNNs learn to accurately predict these dynamics from collected data. Linear and sparse regression methods that used the same set of features as the DNNs were used during early model development phases, but similar to the analytical regression model, the results from these models were not accurate.

The performance of the analytical regression model could be improved (e.g., adding ankle angular velocity and acceleration-dependent terms); however, further system identification would be time-intensive compared to the proposed deep learning pipeline. Additionally, it is difficult to derive analytical models based on a limited set of system states and adding more sensors (e.g., wearables) could cause discomfort to the users or hinder their mobility. The deep learning approach presented in this research provides accurate predictions, eliminates the need to construct analytical models, and only requires a small set of input features.

The results from this research suggest that high fidelity prediction models can be constructed with minimal sensor inputs. If implemented with wearable sensors, these prediction models would obviate the need for vision systems or electromyography sensors to produce accurate predictions during steady-state walking. For future work, one way to distinguish the capabilities of each DNN would be to analyze each for their dependence on data. For instance, one architecture may perform better with fewer training data than others. This has important implications for clinical applications since it would reduce the amount of time to complete data collections, the number of clinical visits, and number of walking trials.

The use of a single experimental condition (i.e., steady-state walking) is also a limitation of this study. Additional training data collection and future tests should include nonrhythmic movements and other tasks, such as stair or ramp walking. This could help determine if some DNN architectures perform better than others across various activities and situations (i.e., generalize to different torque profiles). In this study, the DNNs all performed similarly for steady-state walking conditions. However, GRU and DA-GRU, which include memory-based mechanisms in their model architectures, could better anticipate gait variations in non-steady state situations. The PAFP controller could then pre-actuate the prosthesis in anticipation of these events.

A limitation of this experiment was that model predictions were conducted offline. Real-time tests on actual hardware should also be conducted as future work in order to test the feasibility of evaluating these models in real time for predictive control of robotic prostheses. These tests would also give insight into prediction latency and the effects of sensor noise variations. The filtering techniques must also be addressed in future studies that focus on the implementation of these models towards online applications. One option is to use wearable sensors that include on-board filtering algorithms. Furthermore, an instrumented pyramid adapter (e.g., [42]) and thigh-mounted IMU could be integrated into the PAFP system to provide real-time measurements of the prosthesis-side vertical GRF and hip angles, respectively. An instrumented insole could be used on the intact limb to output load rate measurements in real time and also inform the DNN models if the user is in double- or single-leg support.

Only a single participant was included in the training data, which is an inherent limitation of this study. The focus of this research was to create predictive models that are personalized to the individual, but more subjects should be included when training future models to see if this strategy translates across individuals or if there are any benefits to utilizing inter-user models. Anthropometric and subject-specific data (e.g., weight, height, gender, or limb lengths) could be used as additional features to possibly create models that generalize across individuals. Even if the models need to be retrained for each device and individual, the same set of features and neural network architectures would likely achieve a similar performance for other devices and individuals. Finally, improvements to the inverse dynamics model can be made (e.g., accounting for the elastic behavior of the foot keel, using the PAFP mass as a model parameter, and setting a custom ankle joint center for the PAFP).

## 5. Conclusions

This research aims to take strides toward model-based control methods for PAFPs by developing predictive DNN models. Ambulation data from a single subject were used to train the analytical regression model and three DNN architectures. The trained DNNs demonstrate that sophisticated and high-fidelity models can be used to accurately predict total ankle torque values that are normally only available after computing full-body inverse dynamics offline. Performance was within 5% of the total range of PAFP torque values for both one-sample- and twenty-sample-ahead (half a gait cycle) predictions. The results suggest these models can be used to estimate dynamics that are typically unobservable in real time. Thus, these human–robot predictive models could enable model-based powered prosthesis control strategies, which have the potential to improve the functionality of powered prosthetic devices.

## Figures and Tables

**Table 1:** Hyperparameter values tested for optimal performance of the DNNs.

Hyperparameter	Range/Values	Optimal Value					
		1-Sample			20-Sample		
		FFN	GRU	DA-GRU	FFN	GRU	DA-GRU
Sequence Length	[2, 3, 4, … , 18, 19, 20]	16	15	18	20	18	20
Number of Layers	[1, 2, 3]	3	2	-	3	2	-
Number of Hidden Units	[16, 32, 64, 128, 256, 512]	512	512	-	512	64	-
Number of Encoder Hidden Units	[16, 32, 64, 128, 256, 512]	-	-	128	-	-	512
Number of Decoder Hidden Units	[16, 32, 64, 128]	-	-	16	-	-	64
Dropout Probability	[0.1:0.5]	0.114	0.199	-	0.100	0.121	-
Batch Size	[16, 32, 64, 128, 256]	16	16	16	16	16	16
Initial Learning Rate	[$10^{-5}$:$10^{-1}$]	$5.1\times 10^{-5}$	$1.3\times 10^{-4}$	$2.8\times 10^{-3}$	$4.6\times 10^{-5}$	$4.5\times 10^{-4}$	$1.8\times 10^{-3}$
Weight Decay Coefficient	[$10^{-5}$:$10^{-1}$]	0.062	0.019	$3.1\times 10^{-3}$	0.073	0.013	0.070
Learning Rate Reduction Factor	[0.1:0.9]	0.168	0.559	0.132	0.373	0.728	0.230

**Table 2:** Model prediction accuracy measures for full gait cycles.

	RMSE ${}^{1}$ (Nm/kg) ${}^{3}$		% RMSE ${}^{1}$		PCC ${}^{2}$	
	**1-Sample**	**20-Sample**	**1-Sample**	**20-Sample**	**1-Sample**	**20-Sample**
**Analytical**	0.347 ± 0.534	-	26.6 ± 40.9	-	0.822 ± 0.202	-
**FFN**	0.036 ± 0.024	0.058 ± 0.041	2.7 ± 1.6	4.3 ± 2.8	0.996 ± 0.006	0.988 ± 0.019
**GRU**	0.042 ± 0.025	0.068 ± 0.042	3.2 ± 1.7	5.1 ± 3.0	0.995 ± 0.007	0.985 ± 0.024
**DA-GRU**	0.037 ± 0.024	0.058 ± 0.051	2.8 ± 1.5	4.3 ± 3.5	0.996 ± 0.006	0.985 ± 0.030

Mean values ± standard deviations. ^1^ RMSE = Root Mean Squared Error. ^2^ PCC = Pearson Correlation Coefficient. ^3^ Nm/kg = Newton-meter per kilogram bodyweight.

**Figure A1:**
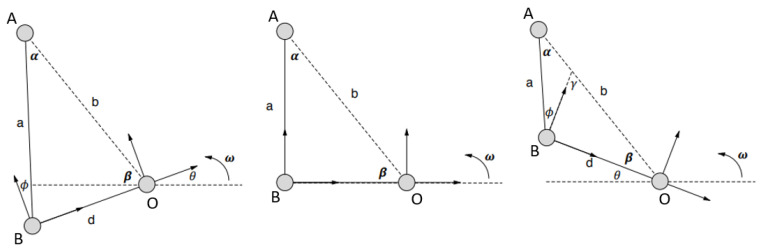
The prototype PAFP represented as two linkages connecting the drivetrain to the ankle joint. Pin joint A is where the ball screw connects to the shank link, pin joint B is where the ball screw nut housing connects to the ankle link, and pin joint O is where the ankle joint is connected to the drivetrain. Lowercase letters represent lengths and Greek letters represent angles. The variable ω represents the direction of positive angular velocity about the ankle joint.

## Data Availability

Data are available by request.

## Appendix A. Derivation of the Analytical Regression Model

For the prototype PAFP system used in this study, the total prosthetic ankle torque $\tau_{p}$ is the sum of the passive torque from the parallel elastic element during loading $\tau_{l}$ and the active torque generated by the motor $\tau_{a}$:

(A1) $$\tau_{p}\left(t\right)=\tau_{a}\left(t\right)+\tau_{l}\left(t\right)$$

The motor is placed parallel to the shank, and as it rotates, the ball screw placed below the motor moves linearly, which creates a translational force on pin B and a moment about pin O (see Figure A1). This moment is the active torque $\tau_{a}$ generated by the motor and is defined as:

(A2) $$\tau_{a}=rF_{s}$$

where $F_{s}$ is resultant linear force of the ball screw acting at joint B and *r* is the moment arm.

Figure A1 illustrates the geometry between the PAFP system linkages for various orientations and shows that lengths *b* and *d* are fixed due to the constant moment arms from pin joint B to pin joint O and the rigid shank link. Length *a*, angle β, angle α, and angle ϕ are all functions of the ankle joint angle θ. Using the positioning of the components, these variables can be defined as follows:

(A3) $$\begin{array}{c} \beta =\arccos\left(\frac{d}{b}\right)+\theta -\theta_{0}\\ a=\sqrt{b^{2}+d^{2}-2bd\cos\left(\beta +\theta -\theta_{0}\right)}\\ \alpha =\arcsin\left(\frac{d\sin\left(\beta \right)}{a}\right)\\ \phi =\frac{\pi }{2}-\beta -\alpha \end{array}$$

where $\theta_{0}$ is an angular offset to ensure that at maximum torque, the ball screw *a* and ankle link moment arm *d* are perpendicular. Using these definitions, the moment arm reduction from pin joint B and pin joint O is:

(A4) $$\frac{\tau_{a}}{F_{s}}=d\cos\left(\phi \right)$$

Using the law of conservation of energy and the principles of screw theory, the mechanical advantage of using the ball screw is defined as:

(A5) $$\frac{\tau_{g}}{F_{s}}=\frac{l}{2\pi \eta_{s}}$$

where $\tau_{g}$ is the torque outputted by the transmission, $\eta_{s}$ is the ball screw efficiency, and *l* is the screw lead (i.e., the linear distance the screw travels in one revolution). Additionally, at the transmission output shaft $\tau_{g}$ and the motor torque $\tau_{m}$ are related by:

(A6) $$\frac{\tau_{g}}{\tau_{m}}=R_{g}\eta_{g}$$

where $R_{g}$ is the planetary gearhead reduction and $\eta_{g}$ is the gearhead efficiency. Using Equations (A4)–(A6), the total mechanical advantage from the motor to the ankle joint is defined as:

(A7) $$\frac{\tau_{m}}{\tau_{g}}\frac{\tau_{g}}{F_{s}}\frac{F_{s}}{\tau_{a}}=\frac{1}{R_{g}\eta_{g}}\frac{l}{2\pi \eta_{s}}\frac{1}{d\cos\left(\phi \right)}$$

Equation (A7) is simplified and the results are inverted as follows:

(A8) $$R_{\tau }=\frac{\tau_{a}}{\tau_{m}}=\frac{d\cos\left(\phi \right)2\pi \eta_{s}\eta_{g}R_{g}}{l}$$

where $R_{\tau }$ is the drivetrain ratio (effective transmission) that relates the applied motor torque to the resultant active ankle torque component. The gain from the motor current command $i_{a}$ to the active torque can be expressed as:

(A9) $$\tau_{a}=R_{\tau }k_{\tau }i_{a}$$

where $k_{\tau }$ is the rated motor torque constant provided by the motor manufacturer.

The passive PAFP elements (e.g., cam device and spring) were designed to be approximated using a third-degree polynomial function [31]. Note that the damping and inertial effects of the motor are assumed to be minimal and are, therefore, not included in the analytical model. The analytical regression problem is formulated as:

(A10) $$\begin{array}{cc} \tau_{p}-\tau_{a} & =\tau_{l}\\ \tau_{p}-R_{\tau }\left(\theta \right)k_{\tau }i_{a} & =p_{3}\theta^{3}+p_{2}\theta^{2}+p_{1}\theta +p_{0} \end{array}$$

where $R_{\tau }\left(\theta \right)$ is a function of the prosthetic ankle angle θ. Additionally, the prosthetic ankle angle θ is the output of the PAFP-mounted encoder sensor. Similarly, the motor current command $i_{a}$ is sampled by the embedded system. Coefficients $p_{0}$, $p_{1}$, $p_{2}$, and $p_{3}$ represent the parameters of the PAFP’s passive torque function to be solved in this nonlinear least squares problem.
